# Paraganglioma of the Neck: A Rare Case With Spinal Metastasis

**DOI:** 10.1155/2024/2025115

**Published:** 2024-04-26

**Authors:** Sean McCormack, Eyad Hamad, Amar Hamad

**Affiliations:** ^1^Saint James School of Medicine, Cane Hall Road, Arnos Vale, Saint Vincent and the Grenadines; ^2^Northwestern School of Medicine, Chicago, Illinois, USA; ^3^Department of Hemetology/Oncology, Advocate Christ Hospital, Oak Lawn, Illinois, USA

**Keywords:** carotid body paraganglioma, diagnostic imaging, extra-adrenal paraganglioma, multidisciplinary approach, neuroendocrine tumor, nonfunctional paraganglioma, oncology, paraganglioma, radiology

## Abstract

Paragangliomas are rare neuroendocrine tumors that arise from the paraganglia, which are clusters of neuroendocrine cells associated with the autonomic nervous system. These tumors are commonly found in the adrenal medulla but can also occur in other locations outside the adrenal gland. Here, we present a case report of a slow-growing paraganglioma in the left neck with spinal metastasis in a 60-year-old man. This case highlights the importance of considering paraganglion tumors in the differential diagnosis of neck masses and the need for early diagnosis and management to prevent potential complications. Importantly, both the clinical picture and anatomical location of these tumors is important when determining treatment plans.

## 1. Introduction

Paragangliomas are rare neuroendocrine tumors that originate from the neural crest cells of the autonomic nervous system. Neuroendocrine tumors are commonly found in the adrenal medulla but can also occur in other locations outside of the adrenal gland. Extra-adrenal neuroendocrine tumors can affect any of the autonomic paraganglia. When these tumors are found in the adrenal medulla, they are called pheochromocytoma. When found in extra-adrenal autonomic paraganglia, these tumors are called paragangliomas. The location of the lesion is the distinguishing factor between pheochromocytoma and paraganglioma as the two are indistinguishable at the cellular level [1–4]. Paragangliomas can derive from either parasympathetic or sympathetic paraganglia. The incidence is similar for both types of paraganglia [5].

Sympathetic paragangliomas arise outside of the adrenal gland anywhere along the paravertebral sympathetic chain. This chain of ganglia spans from the bladder to the base of the skull. Most sympathetic paragangliomas are found in the abdomen, but many can also be found in the neck region.

Parasympathetic paragangliomas are associated with the parasympathetic nervous system. Common locations for these tumors include the aortic arch, the carotid body, and along parasympathetic cranial nerves extending as far cranially as the middle ear [5–7].

Many of these neuroendocrine tumors often secrete catecholamines. The effects of catecholamines can include symptoms associated with increased sympathetic tone, such as hypertension, heart rate, and sweating (sympathetic paragangliomas). However, not all tumors secrete catecholamines. Parasympathetic paragangliomas are not associated with catecholamine secretion. Nonsecretory neuroendocrine tumors lack side effects associated with catecholamine release, including increased sympathetic tone. Instead, they are often limited to the mass effect of the lesion [3, 6–8].

Most paragangliomas are sporadic tumors, but about 30%–40% come from inherited syndromes [9]. Examples include multiple endocrine neoplasia types 2A and 2B (MEN2), neurofibromatosis type 1 (NF1), von Hippel-Lindau (VHL) disease, and the Carney-Stratakis dyad. Genetic screening can be offered for patients diagnosed with a paraganglioma. However, since most patients are diagnosed after the fifth decade of life, this has little effect on decisions about family planning [2, 3, 5, 6, 8].

Paragangliomas are very rare neoplasms. The incidence of pheochromocytoma and paraganglioma ranges between 2 and 10 per million [10, 11]. Of these neuroendocrine tumors, 80%–85% are pheochromocytomas (intra-adrenal) and 15%–20% are paragangliomas (extra-adrenal) [9–11]. The majority of paragangliomas are benign. Malignant paragangliomas are much less common [7]. It was estimated that the incidence of malignant paraganglioma in the United States is only 93 cases per 400 million persons [5]. However, many asymptomatic and slow-growing tumors may go undiagnosed [12]. Here, we present a case report of a slow-growing paraganglioma in the left neck with spinal metastasis of a 60-year-old man.

## 2. Case Presentation

A 60-year-old man presented to our clinic with a slowly growing mass in his left neck. He reported that the mass had been present for at least 30 years. It had never caused him any discomfort, and it had never been fully evaluated. Recently, the patient had noticed a change in taste and two swollen supraclavicular lymph nodes which prompted him to seek evaluation. Physical examination revealed a firm, nontender mass that measured approximately 3 cm in diameter in the left neck, at the level of the carotid bifurcation. There were two associated swollen supraclavicular lymph nodes inferior to the mass. These lymph nodes were firm and nontender. No other lymphadenopathy or other palpable masses were observed on examination. The patient denied fatigue, weight changes, loss of appetite, neck pain, back pain, palpitations, hot flashes, or other constitutional symptoms.

**Figure 1:**
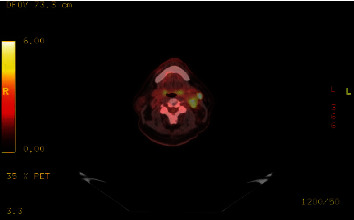
A hypermetabolic heterogeneous soft tissue mass at the left carotid bifurcation demonstrates SUV max of 7.1. This lesion is consistent with the given history of biopsy-proven paraganglioma. Multiple hypermetabolic left cervical lymph nodes are also seen (SUV max of 16.1).

**Figure 2:**
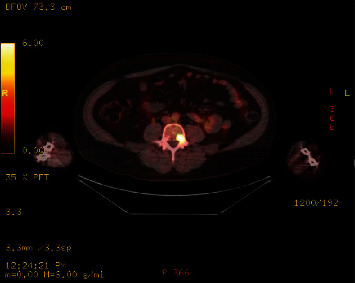
Increased uptake seen in the left L4 pedicle measuring 1.8 cm × 1.2 cm demonstrates SUV max of 6.8. This lesion is consistent with biopsy-proven osseous metastatic disease.

Further investigations were carried out, including a computed tomography (CT) scan, magnetic resonance imaging (MRI) of the neck, and full body positron emission tomography (PET). The CT scan revealed a well-defined hypervascular mass in the left neck, measuring 2.5 × 3 cm in size, at the level of the carotid bifurcation. The MRI showed a T1 hypointense and T2 hyperintense mass, consistent with a paraganglioma surrounding the carotid artery. Enlarged supraclavicular lymph nodes were also observed. The PET scan showed intense fluorine-18-labeled fluorodeoxyglucose (18F-FDG) uptake in the neck lesion and lymph nodes (Figure 1). There was also increased 18F-FDG uptake within the L4 vertebral pedicle, consistent with metastatic disease (Figure 2). No other areas of abnormal 18F-FDG uptake were noted. Further biochemical investigations were carried out, including measurements of plasma and urinary catecholamines and metanephrines, which were within normal limits.

A biopsy of the mass was performed that confirmed the diagnosis of paraganglioma. A biopsy of the spinal lesion was also performed confirming metastatic disease. The patient was referred for an interdisciplinary team that included a medical oncologist, radiation oncologist, and an otolaryngological surgeon. The patient was presented with the prognosis, disease course, and treatment options. Shared decision-making with the patient was used to decide treatment. While the patient was not a good surgical candidate due to the paraganglioma being at the carotid bifurcation, multiple other treatment options including radiation, chemotherapy, and immunotherapy were proposed. Ultimately, it was decided that due to the slow progression of the disease over the past 30 years and the lack of symptoms, close observation was the patients' preferred course of action. The patient was provided with education on paragangliomas and worsening symptoms and scheduled for close follow-up. The patient was advised that his children receive genetic testing to screen for disease and to aid in decisions regarding family planning. Benefits of early detection were described.

## 3. Discussion

Paragangliomas are rare tumors that arise from paraganglia, which are clusters of neuroendocrine cells associated with the autonomic nervous system. These tumors are usually benign but can be malignant in a small proportion of cases. Paragangliomas can occur in various locations, including the head and neck, chest, abdomen, and pelvis. The location of the tumor affects its symptoms, treatment, and prognosis. For instance, a paraganglioma in the head and neck region can cause difficulty swallowing, hoarseness, or changes in facial sensation, while a tumor in the abdomen can lead to abdominal pain and weight loss [6, 7].

When a patient is suspected to have a paraganglioma, a biochemical workup is required. Biochemical investigations, such as measuring metabolites of epinephrine and norepinephrine, metanephrines, and normetanephrines, are useful in coming to the diagnosis if the tumor is a secretory type. Measuring for free catecholamine excess can also lead you to the diagnosis; however, measuring for metabolites has been shown to be a superior diagnostic test. Clinical features of catecholamine excess are high blood pressure, palpitations, and sweating. These symptoms can be challenging to diagnose and manage, as they are often nonspecific and can mimic many other medical conditions. Biochemical investigations can help distinguish plasmacytoma from other diseases [9].

Genetic sequencing can be of use if a person is diagnosed with paraganglioma. Many paragangliomas are associated with genetic syndromes, while others occur sporadically. As we have learned more about these tumors, it has been shown that up to 40% arise from an attributable germline mutation. Understanding the genetic basis of paragangliomas is crucial for early detection and management of these tumors, especially in people with children at risk or a positive family history. Paragangliomas can be associated with inherited syndromes, and genetic screening is recommended to all patients with pheochromocytomas and paragangliomas [9]. Common reasons individuals decide against genetic testing include the emotional distress it may cause, fearing the implications of learning about their predisposition to inheritable diseases. Additionally, concerns about the lack of effective treatments or outcomes, coupled with worries about privacy, cost, ethical beliefs, and the potential for false reassurance from negative results, contribute to their decision to forgo testing. Our patient decided to forgo testing, and his adult children were informed to make their own educated decision.

Paragangliomas can be diagnosed by a variety of imaging studies, both when the tumor is suspected and as an incidental finding. This is due to the indolent course of many of these tumors. Anatomical imaging using technology, such as CT or MRI, can detect masses such as paragangliomas. These findings need to be confirmed by biopsy. Functional imaging using tracers such as 123I-metaiodobenzylguanidine (MIBG) is both highly sensitive and specific for intra-adrenal pheochromocytomas. This has led to its use as the primary functional imaging method. However, its shortfall is its low sensitivity for extra-adrenal tumors. PET is useful for diagnosing paragangliomas, as is seen in this case report. A variety of radiotracers have been evaluated in patients with either pheochromocytomas or paragangliomas. PET imaging using 18F-FDG or 68Ga-labeled somatostatin analogs (68Ga-DOTA-TOC/TATE/NOC) is especially useful for investigating if metastatic lesions are present [9]. Somatostatin receptors are often expressed in neuroendocrine tumors, especially low-grade tumors. However, the expression of somatostatin receptors and thus the uptake generally decrease in both moderate- and high-grade tumors. The result is usually the reverse for metabolic imaging with 18F-FDG. Lesions generally have more 18F-FDG uptake with increasing proliferation. For visualization of higher-grade neuroendocrine tumors, 18F-FDG may be the preferred imaging modality [13–15]. 18F-FDG was used in our patient due to his high-grade lesion. Further studies have shown the patients undergoing imaging with both 18F-FDG and somatostatin receptor imaging show the results to be complementary and increase the sensitivity [13–16]. Because of financial restraints, this optimal dual nuclear imaging strategy is not applied at all centers.

The management of paragangliomas depends on the location, size, and extent of the tumor, as well as the presence of metastasis [17]. Surgical resection is usually the first-line treatment for many paragangliomas and can be curative in nonmetastatic disease [1, 18]. In some cases, radiation therapy, injection ethanol ablation, and chemotherapy may also be used or even preferred [19–21], particularly in cases where the tumor is malignant or difficult to resect. In metastatic disease, however, there is no cure, and because all treatment options carry risk, in patients with indolent disease, the best treatment may simply be observation with periodic biochemical testing and imaging [22].

While our patient decided to forgo surgical intervention at this time, patients that wish to be treated with surgery must be treated with care. Premedication prior to surgery is often necessary for patients with paragangliomas due to the risk of catecholamine release during surgical manipulation of the tumor. Catecholamines are hormones produced by paragangliomas that can cause significant increases in blood pressure, heart rate, and cardiac output. These effects can be life-threatening in some cases and require careful management. The premedication regimen typically includes medications that block the effects of catecholamines, such as alpha-blockers followed by beta-blockers. Alpha-blockers work by blocking the action of epinephrine and norepinephrine in blood vessels, thus relaxing the smooth muscles in blood vessels, causing vasodilation. This is effective at reducing blood pressure and reducing the risk of a hypertensive crisis. Beta-blockers, on the other hand, block the action of epinephrine and norepinephrine on the heart, reducing heart rate and cardiac output [23].

The timing is crucial, as drug delivery should be administered several days before surgery to allow for adequate alpha-blockade thus preventing sudden blood pressure surges during tumor manipulation. An optimal duration of preoperative treatment has not been described, but a minimum of 7 days is advised [23]. In some cases, patients may also require additional intravenous medications to manage any acute hypertensive crises during surgery. A thorough evaluation and management plan should be tailored to each patient's specific needs to optimize outcomes and minimize complications [18, 23, 24]. Accurate assessment of tumor functionality, specifically regarding their ability to synthesize, store, and potentially secrete catecholamines, is crucial for determining the necessity of preoperative *α*-adrenoceptor blockade. Even small tumors or those with normal biochemical test results can cause dangerous spikes in blood pressure. The increasing misuse of the term “nonfunctional” may mislead individuals into assuming that adrenoceptor blockade is unnecessary. “Nonfunctional” tumors can neither synthesize nor secrete catecholamines. It is important to differentiate nonfunctional tumors from tumors that are nonsecretory before deciding to forgo blockade prior to surgery [25].

## 4. Conclusion

We have presented a case of a slow-growing carotid body paraganglioma in the left neck of a 60-year-old man. Paragangliomas are rare neuroendocrine tumors that arise from the neural crest cells of the autonomic nervous system. Multiple types of paragangliomas exist: sympathetic, parasympathetic, secretory, and nonsecretory types. As these different types of lesions can occur at many different locations, the potential symptoms have significant variance in how they present symptomatically. While the case presented had very little symptoms, other patients with paragangliomas may present with many symptoms. Due to the rarity of this disease, expertise in this condition is extremely rare. Using an interdisciplinary team can improve the management and outcomes in patients with this condition. This case highlights the importance of considering paragangliomas in the differential diagnosis of neck masses, and the importance of early diagnosis and management to prevent potential complications. Here, we hope to increase awareness of this disease so others consider this a possibility when diagnosing a patient that presents with a neck mass that may or may not be symptomatic. There is a need for continued awareness of paragangliomas to better recognize and treat patients with these rare tumors.

## Data Availability

Data sharing is not applicable to this article.
